# The long noncoding RNA *LUCAT1* promotes colorectal cancer cell proliferation by antagonizing Nucleolin to regulate MYC expression

**DOI:** 10.1038/s41419-020-03095-4

**Published:** 2020-10-23

**Authors:** Runliu Wu, Liang Li, Yang Bai, Bowen Yu, Canbin Xie, Hao Wu, Yi Zhang, Lihua Huang, Yichao Yan, Xiaorong Li, Changwei Lin

**Affiliations:** 1 Department of Gastrointestinal surgery, The Third XiangYa Hospital of Central South University, Changsha, Hunan 410013 China; 2 Class 25 grade 2016, The Five-Year Program in Clinical Medicine, School of Medicine, University of South China, Hengyang, Hunan 421001 China; 3 Center for Experimental Medicine, The Third XiangYa Hospital of Central South University, Changsha, Hunan 410013 China; 4 Department of Gastroenterological Surgery, Peking University International Hospital, Beijing, 102206 China; 5 School of Life Sciences, Central South University, Changsha, Hunan 410078 China

**Keywords:** Colorectal cancer, Colorectal cancer

## Abstract

The long noncoding RNA (lncRNA) *LUCAT1* was recently reported to be upregulated and to play an essential role in multiple cancer types, especially colorectal cancer (CRC), but the molecular mechanisms of *LUCAT1* in CRC are mostly unreported. Here, a systematic analysis of *LUACT1* expression is performed with data from TCGA database and clinic CRC samples. *LUCAT1* is identified as a putative oncogene, which is significantly upregulated in CRC and is associated with poor prognosis. Loss of *LUCAT1* restricts CRC proliferative capacities in vitro and in vivo. Mechanically, NCL is identified as the protein binding partner of *LUCAT1* by using chromatin isolation by RNA purification coupled with mass spectrometry (ChIRP-MS) and RNA immunoprecipitation assays. We also show that NCL directly binds to *LUCAT1* via its putative G-quadruplex-forming regions from nucleotides 717 to 746. The interaction between *LUCAT1* and NCL interferes NCL-mediated inhibition of MYC and promote the expression of MYC. Cells lacking *LUCAT1* show a decreased MYC expression, and NCL knockdown rescue *LUCAT1* depletion-induced inhibition of CRC cell proliferation and MYC expression. Our results suggest that *LUCAT1* plays a critical role in CRC cell proliferation by inhibiting the function of NCL via its G-quadruplex structure and may serve as a new prognostic biomarker and effective therapeutic target for CRC.

## Introduction

Colorectal cancer (CRC) is the third most common malignancy and the third leading cause of cancer-related death worldwide^1,2^. The CRC morbidity and mortality rates in China have steadily increased^3^. Therefore, it is a priority to increase our understanding of the mechanisms that drive CRC progression and to identify diagnostic and therapeutic targets to treat CRC.

Long noncoding RNA (lncRNA) is defined as noncoding RNA (ncRNA) sequences that are longer than 200 nucleotides^4^. Accumulating evidence indicates that lncRNAs play critical roles in multiple cellular and biological processes, including cell development, differentiation, growth, tumorigenesis, and metastasis^5,6^. LncRNAs execute their functions in the cytoplasm or nucleus through diverse mechanisms, such as protein binding, chromatin modification, cytoplasmic scaffolding, and RNA decay, acting as regulators of gene expression^7–10^. To date, several important lncRNAs, such as CRNDE^11^, SNHG7^12^, and lncRNA-FEZF1-AS1^13^, have been shown to participate in CRC development and can be regarded as potential, new diagnostic, and therapeutic targets. Lung cancer-associated transcript 1 (*LUCAT1*), also known as *SCAL1*, was first identified in smoking-induced lung cancer cells^14^. *LUCAT1* dysregulation was observed in various cancers, including non-small-cell lung cancer, glioma, renal cell carcinoma, esophageal squamous cell carcinoma, prostate cancer, cutaneous squamous cell carcinoma, and CRC^15–21^. However, the specific function of *LUCAT1* and its regulatory mechanism in CRC proliferation remain largely unknown.

Nucleolin (NCL) is one of the most multifunctional RNA-binding proteins (RBPs). It is most abundant in the nucleolus and has been demonstrated to contribute to G-quadruplex (G4) formation in the promoter regions of some oncogenes. Studies have demonstrated that G4 is a negative regulator of transcription^22^. Small RNAs have been shown to be involved in changes in the G4 structure or to act as molecular decoys for G4-binding proteins, altering gene expression, or inhibiting protein activity^23,24^. However, studies touching upon whether lncRNAs can regulate the G4 formation of oncogenes are rarely reported, and it is unclear whether *LUCAT1* could function along with NCL to regulate G4-associated gene expression and CRC progression.

In this study, we found that *LUCAT1* upregulation in CRC leads to cancer cell proliferation. The colocalization and binding of *LUCAT1* and NCL suggest that *LUCAT1* plays a regulatory role by antagonizing the function of NCL, leading to an increase in MYC expression. Overall, our results provide new evidence that *LUCAT1* exerts an oncogenic function, and the *LUCAT1*/NCL/MYC axis might be a potential prognostic marker and therapeutic target for CRC.

## Materials and methods

### Cell lines and cell culture

SW620 (RRID: CVCL_0547) and SW480 (RRID: CVCL_0546) cells were cultured in L15 (KeyGEN BioTECH, Jiangsu, China) medium supplemented with 10% fetal bovine serum (FBS, Biological Industries, Israel) and 1% antibiotics (100 U/ml penicillin and 100 mg/ml streptomycin; Life Technologies, Inc., Grand Island, NY, USA) and cultured at 37 °C in 5% CO_2_. SW480 (primary colon adenocarcinoma) and SW620 (subsequent lymph node metastasis) cells were derived from the same patient. HCT116 (RRID: CVCL_0291), HT29 (RRID: CVCL_0320), and NCM460 (RRID: CVCL_0460) cells were cultured in McCoy’s 5 A (KeyGEN BioTECH) medium supplemented with 10% FBS and 1% antibiotics and cultured at 37 °C in 5% CO_2_. All cell lines were obtained from KeyGEN BioTECH (Jiangsu, China). All cell lines have been authenticated using STR (or SNP) profiling within the last 3 years. All experiments were performed with mycoplasma-free cells.

### Patients and tissue sampling

CRC tissues and adjacent normal tissues used were obtained from the 36 patients who underwent surgical resection for CRC at the Third XiangYa Hospital of Central South University (Changsha, China) after informed consent was obtained. The study was approved by the ethics committee of the Third XiangYa Hospital of Central South University (No. 2016-S086). Before the surgical resections, no preoperative treatment was administered. Tissue specimens were collected during surgery and immediately stored in liquid nitrogen. All tissue samples were diagnosed and confirmed by a pathologist.

### Lentivirus infection and transfection

Lentiviruses, including Cas9, sgRNA, and scramble (negative control) for *LUCAT1*, were obtained from GeneChem (Shanghai, China) for in vivo experiments. Cells were seeded at 60–70% confluence and infected with lentivirus culture (at an MOI of 10). The medium was replaced with fresh medium containing puromycin after 24 h, and noninfected cells were eliminated with puromycin. To generate stable lentivirus-transduced lines, cells were infected with virus and polybrene following the manufacturer’s recommendations, and stable cell lines were selected with 4 µg/ml puromycin treatment after 72 h of transfection. The efficiency in different cells was determined by qRT-PCR and Western blot. The shRNAs for NCL were obtained from RiboBio Co., LTD. (Guangzhou, China). The shRNAs were transfected with HiPerFect Transfection Reagent (Qiagen, Hilden, Germany). For each well of a 6-well plate (7 × 10^5^ cells/well), 10 µL of shRNA (20 µM) and an RNAi negative control were diluted in 100 µL of serum-free culture medium, and then 12 µL of HiPerFect Transfection Reagent was added. The mixture was incubated for 10 min at room temperature; then, the mixture was added dropwise onto cells, and the plate was swirled gently. The cells were incubated for an additional 72 h for further analysis. All sequences are provided in Supplementary Table 1.

### Quantitative real-time PCR assays

Total RNA from cells and tissues was extracted using TRIzol Reagent (Invitrogen, Carlsbad, CA, USA) according to the manufacturer’s instructions. cDNA was synthesized using ReverTra Ace qPCR RT Master Mix (TOYOBO, Osaka, Japan). Quantitative real-time PCR (qRT-PCR) was performed on a LightCycler 480 Real-Time PCR instrument (Roche, Basel, Switzerland) using SYBR Green Real-time PCR Master Mix (TOYOBO). Each sample was quantified in triplicate, and the experiment was repeated three times. Analysis was performed using the 2^−∆∆Ct^ method, with GAPDH as the endogenous control. All primer pairs were purchased from Sangon Biotech (Shanghai, China), and sequences are provided in Supplementary Table 2.

### Genomic PCR assays

Genomic DNA was extracted using a QIAamp DNA Mini Kit (Qiagen). PCR was performed on an Applied Biosystems instrument using Taq PCR StarMix (GenStar, Beijing, China). The amplification products were detected using 1% agarose gel electrophoresis and visualized on a Syngene Bio Imaging system. Primer sequences are provided in Supplementary Table 2.

### Cell proliferation and colony formation assays

A cell counting kit 8 (CCK8) assay (Dojindo, Kumamoto, Japan) was used to measure cell proliferation in 96-well plates. Cells were seeded at 4 × 10^3^ or 8 × 10^3^ cells per well, with five replicates for each condition. CCK8 was added at 0, 24, 48, and 72 h and incubated at 37 °C for 2 h. Cell numbers were determined by measuring the absorbance at 450 nm using a 96-well format plate reader (Perkin Elmer, MA, USA). For colony formation assays, 500 cells were seeded in each well of a 6-well plate in triplicate for each condition and incubated for 10 or 14 days. The colonies were fixed with methanol, stained with crystal violet, and counted. The average colony counts were calculated, and a paired *t*-test was used to test statistical significance. Each experiment was repeated three times.

### RNA immunoprecipitation

RIP experiments were carried out with the Magna RIP Kit (Millipore, #17-700, CA, USA) according to the manufacturer’s instructions using 5 µg of a rabbit anti-NCL antibody (Cell Signaling Technology, MA, USA, #14574) or rabbit IgG. Proteins isolated from beads were analyzed by Western blot. The coprecipitated RNAs were detected by real-time PCR.

### Chromatin isolation by RNA purification (ChIRP) assays

ChIRP experiments were performed using the Magna ChIRP RNA Interactome Kit (Millipore, #17-10494) according to the manufacturer’s protocol. The cells were crosslinked using 1% glutaraldehyde and then sonicated at high intensity 40 times for 15-s pulses and 1-min intervals at 4 °C. The lengths of the sheared DNA were confirmed by 1% agarose gel electrophoresis analysis. The probes (100 pmol) for *LUCAT1* were used for hybridization with sonicated cell lysate and streptavidin-coated magnetic beads. For RNA isolation, TRIzol Reagent and a Qiagen miRNeasy Mini Kit (Qiagen) were used. DNA isolation was performed according to a standard protocol. For protein collection, 3% formaldehyde was used for crosslinking, and the isolated proteins were washed and heated several times. The proteins were analyzed by Western blot. Probe sequences are provided in Supplementary Table 3.

### RNA fluorescence in situ hybridization (FISH) and immunofluorescence

The cell smears were prepared for FISH using the standard methods. After fixation with 4% formaldehyde at room temperature for 10 min, the slides were permeabilized with 0.5% Triton X-100 at 4 °C for 10 min. The slides were incubated overnight at 37 °C in hybridization solution with the FISH probes. After hybridization, the slides were washed with wash buffer I (4 × SCC, 0.1% Tween-20), wash buffer II (2 × SCC), wash buffer III (1 × SCC) and PBS for 5 min at 42 °C. The slides were stained with 1 mg/ml 4′,6-diamidino-2-phenylindole (DAPI) for 10 min and then washed three times for 3 min. For colocalization studies, after RNA FISH, immunofluorescence was performed according to a standard protocol. A rabbit anti-NCL antibody (Cell Signaling Technology, #14574, 1:100) was used to detect the colocalization of *LUCAT1* with NCL in CRC cells. Images were taken with a microscope (Olympus). All experiments were repeated three times. All FISH probes were commercially synthesized by RiboBio Co., LTD.

### Western blot assays

Whole-cell lysates were prepared in 1 × RIPA buffer (KeyGEN) containing 1% PMSF (KeyGEN). The proteins were separated by sodium dodecyl sulfate-polyacrylamide gel electrophoresis (SDS–PAGE), transferred to polyvinylidene fluoride membranes (Millipore), blocked with 5% skim milk for 1 h at room temperature, immunoblotted with primary antibodies overnight and secondary antibodies for 1 h, and visualized on an Odyssey CLx Infrared Imaging System (LI-COR Biosciences, NE, USA). Antibodies used for Western blot analysis are provided in Supplementary Table 4.

### Chromatin immunoprecipitation (ChIP) assays

ChIP assays were carried out using the EZ-ChIP Kit (Millipore, #17-371) according to the manufacturer’s protocol. Briefly, the cells were grown to 90% confluence in a 150 cm^2^ culture dish and crosslinked with 1% formaldehyde at room temperature for 10 min. Glycine (10×) was used to quench excess formaldehyde, and the cells were scraped and pelleted. Chromatin in lysis buffer was sonicated on ice to 200-1000 bp with a Vibra-Cell sonicator (Sonics & Materials, Inc., CT, USA) at 75% intensity two times (65% once and 55% once for 10-s pulses and 50-s intervals). The sheared DNA lengths were confirmed by agarose gel electrophoresis, and the chromatin was precleared with protein G-agarose beads (50% slurry). Precleared chromatin was incubated with an anti-NCL antibody (Novus Biologicals, CO, USA, NB600-241) or IgG and rotated at 4 °C for 12 h. Elution and reverse crosslinking of the protein-DNA complexes were performed according to the manufacturer’s protocol. Spin columns were used for the ChIP DNA extraction. Standard PCRs were performed, and products were detected using 1% agarose gel electrophoresis and visualized on a Syngene Bio Imaging system. The PCR primers used to amplify the promoter region are provided in Supplementary Table 5.

### Luciferase reporter assays

Cells were seeded at a density of 8 × 10^4^ cells per well in a 24-well plate. Cells were transfected with the reporter plasmid sh-NCL or the corresponding shRNA negative control and the *LUCAT1* overexpression plasmid or the corresponding negative control plasmid by using Lipofectamine 2000 according to the manufacturer’s instructions (Invitrogen). The Renilla luciferase sequence in the pRL-TK vector (Promega, WI, USA) was used as an internal control. The luciferase activity was measured 24 h after transfection by using a Luciferase Assay System (Promega) with a luminometer (Perkin Elmer). The firefly luciferase activity was normalized to the Renilla luciferase activity. The data are expressed as the percent of luciferase activity in control cells (100%). All plasmids were commercially synthesized by RiboBio Co., LTD., and their information is provided in Supplementary Table 9.

### Nude mouse xenograft tumor growth model and immunohistochemistry assays

Experiments with animals were performed in accordance with the guidelines for experimental animal management established by Kagawa University and guidelines for the welfare and use of animals in cancer research^25^. Experiments were carried out under pathogen-free conditions with randomly chosen littermates of the same sex, matched by age and body weight. The health status of mouse lines was routinely checked by veterinary staff. Five-week-old male BALB/c nude mice (nu+/nu+) were obtained from the Department of Laboratory Animals of Central South University. To investigate tumor growth in vivo, 2 × 10^6^ cells were harvested and injected subcutaneously into the left or right flank of nude mice (*n* = 5 per group). After 30 days of the injection, the mice were killed by an overdose of pentobarbital (250 mg/kg, intraperitoneal injection), and the tumors were measured with calipers and an electronic scale to estimate the tumor volume and weight, respectively. The tumor volumes were calculated based on the formula: volume (mm^3^) = length (mm) × width (mm) × width (mm)/2. Tumors were further embedded in paraffin for H&E and IHC.

### Bioinformatics analysis

Gene expression analysis of *LUCAT1* was carried out using the starBase database v3.0 project (http://starbase.sysu.edu.cn)^26^. The survival analysis of *LUCAT1* was carried out using the Gene Expression Profiling Interaction Analysis database (http://gepia.cancer-pku.cn)^27^. The relationship between *LUCAT1* and NCL was predicted with the RNA-Protein Interaction Prediction software (http://pridb.gdcb.iastate.edu/RPISeq/)^28^. The potential binding sites between *LUCAT1* and NCL were predicted with the QGRS Mapper software (http://bioinformatics.ramapo.edu/QGRS/index.php)^29^.

### Statistical analysis

Statistical analysis was performed using GraphPad Prism 7.0 to evaluate differences among experimental groups. Data were presented as mean ± s.d. of three independent experiments except where otherwise indicated. All data met the assumptions of the tests (e.g., normal distribution). Student’s *t*-test was used to analyze the assays. One-way analysis of variance (ANOVA) was used for comparison between the different groups. The relationship between gene expression and clinical pathological indicators is examined through chi-square test. The results with *p* < 0.05 were considered statistically significant. One asterisk, two asterisks, three asterisks and four asterisks indicate *p* < 0.05, *p* < 0.01, *p* < 0.001 and *p* < 0.0001, respectively. No samples or animals were excluded. No statistical methods were used to predetermine sample sizes. Sample sizes were similar to those generally employed in the field.

## Results

### *LUCAT1* is upregulated in CRC and correlates with a poor prognosis

**Fig. 1:**
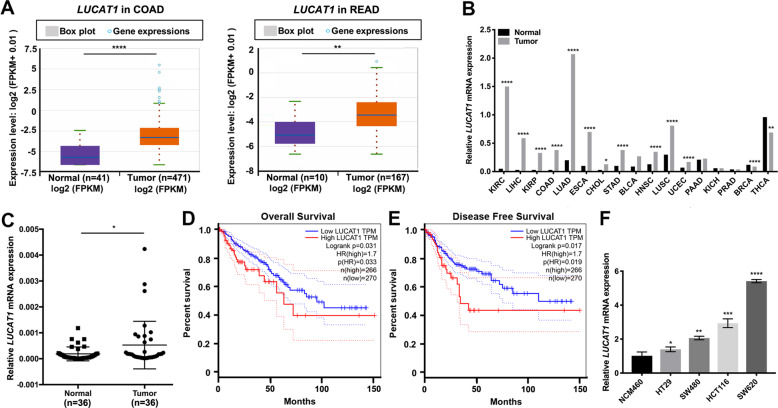
*LUCAT1* is upregulated in CRC tissues. (**A**) *LUCAT1* had higher expression in CRC samples than in matched normal tissues from TCGA database (colon adenocarcinoma (COAD), Normal=41, tumor=471; rectum adenocarcinoma (READ), Normal=10, tumor=167). (**B**) The relative expression level of *LUCAT1* in kidney renal clear cell carcinoma (KIRC), liver hepatocellular carcinoma (LIHC), COAD, lung adenocarcinoma (LUAD), esophageal carcinoma (ESCA), stomach adenocarcinoma (STAD), head and neck squamous cell carcinoma (HNSC), lung squamous cell carcinoma (LUSC), and uterine corpus endometrial carcinoma (UCEC). (**C**) *LUCAT1* in CRC tissues was upregulated compared with that in adjacent normal tissues, *n* = 36 (measured by qRT-PCR; GAPDH was used as an internal control). (**D**) Kaplan–Meier estimated overall survival in patients with high or low *LUCAT1* expression, higher *LUCAT1* expression with poorer overall survival, *p* = 0.031. Group cutoff-point: 25% (high) and 75% (low). (**E**) Kaplan–Meier estimated disease-free survival in patients with high or low *LUCAT1* expression, higher *LUCAT1* expression with poorer disease-free survival, *p* = 0.017. Group cutoff points were 25% (high) and 75% (low). (**F**) *LUCAT1* expression in colorectal cancer cell lines (HT29, SW480, HCT116, and SW620) was increased compared to that in NCM460 cells, a normal colon cell line (measured by qRT-PCR; GAPDH was used as an internal control). The results are presented as the mean ± s.d. and are representative of at least three independent experiments. *p < 0.05, **p < 0.01, ***p < 0.001, ****p < 0.0001.

XLOC_004924, also named *LUCAT1*, was identified as one of the most significantly upregulated lncRNAs in CRC based on the gene profiling data that we have previously reported^11^. According to the ENSEMBL database, there are 61 transcriptomes of *LUCAT1*, and all isoforms are noncoding RNAs. *LUCAT1*-201 (ENST00000511918.6) was reported to be one of the functional transcripts, and its sequence was consistent with XLOC_004924. For the remainder of the manuscript, all instances where *LUCAT1* is mentioned were *LUCAT1*-201. Next, we found that *LUCAT1* was also highly expressed in CRC according to the data from starBase v3.0 (Fig. 1A). In addition, *LUCAT1* upregulation was also reported in other cancer types (Fig. 1B). To validate this result, we performed qRT-PCR to evaluate the expression level of *LUCAT1* in 36 pairs of CRC and adjacent normal tissues. The results showed that *LUCAT1* expression in cancer tissues was higher than that in adjacent normal tissues in CRC patients (*p* = 0.024, Fig. 1C). Next, we summarized the clinicopathological characteristics of these patients, and the data demonstrated that patients with higher *LUCAT1* expression showed a poorer histopathological grade and more lymph node metastasis than the patients with lower *LUCAT1* expression (Table 1). Additionally, we examined the correlation between the *LUCAT1* expression level and the prognosis of CRC patients through the GEPIA database by using the same cutoff point. Kaplan–Meier survival analysis showed that patients with higher levels of *LUCAT1* had shorter overall survival and disease-free survival times than those who had lower levels of *LUCAT1* (Fig. 1D, E). Moreover, the expression level of *LUCAT1* was detected in 4 human CRC cell lines (HCT116, HT-29, SW620, and SW480) and normal human colonic epithelial NCM460 cells, and the results showed that *LUCAT1* was significantly higher in cancer cell lines than in NCM460 cells (Fig. 1F). HCT116 and SW620 cells with relatively higher *LUCAT1* expression were selected for subsequent functional assays.

**Table 1:** Clinic-pathological characteristics of enrolled patients.

Clinicopathological characteristics	Total (*n* = 36)	*LUCAT1*		
		Low^a^ (*n* = 27)	High^a^ (*n* = 9)	*P*-value
Mean age (years)	60.89 ± 12.331	60.83 ± 2.655	60.94 ± 3.216	0.979
Sex				0.414
Male	24	19	5	
Female	12	8	4	
Tumor site				0.431
Left colon	9	8	1	
Right colon	11	7	4	
Rectum	16	12	4	
Staging				0.695
II	12	9	3	
III	22	17	5	
IV	2	1	1	
Tumor size				0.842
<5 cm	19	14	5	
≥5 cm	17	13	4	
Differentiation				0.033^b^
CRC, WD	7	7	0	
CRC, MD	17	14	3	
CRC, PD	12	6	6	
Tumor stage				0.519
T2	1	1	0	
T3	27	19	8	
T4	8	7	1	
Lymph node metastasis				
N0	13	10	3	
N1	13	12	1	0.022^b^
N2	10	5	5	
Distant metastasis				0.400
M0	34	26	8	
M1	2	1	1	

^a^Low and high expression groups were determined by the cutoff-point 75% (27 of 36) and 25% (9 of 36) of LUCAT1 in 36 tumor tissue specimens.

^b^Statistical significance (*P* < 0.05).

### *LUCAT1* is required for the proliferation of CRC cells in vitro and in vivo

**Fig. 2:**
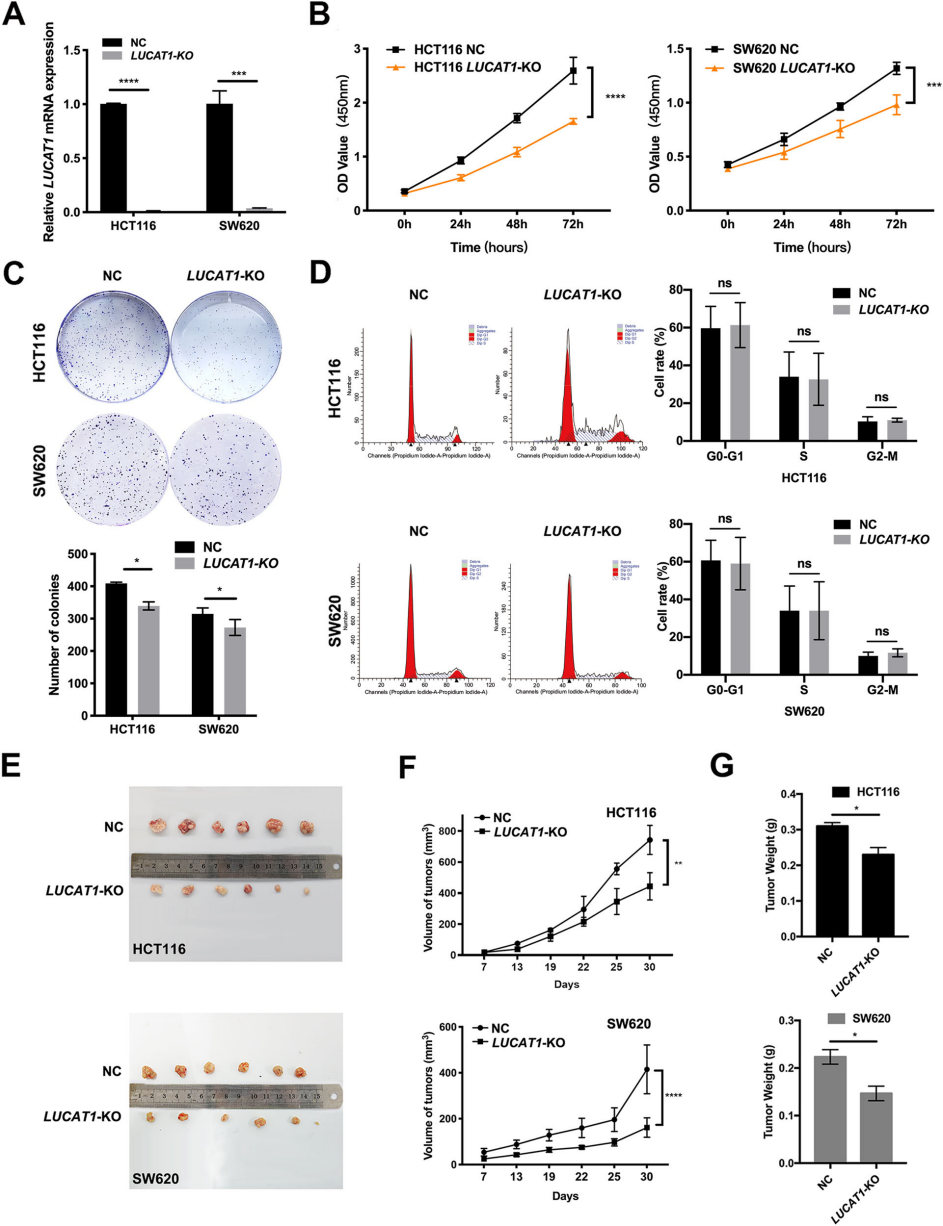
*LUCAT1* promotes CRC cell proliferation in vitro and in vivo. (**A**) The expression of *LUCAT1* was detected by qRT-PCR in HCT116 cells, and cells transfectedwith the CRISPR/Cas9 system differed significantly between the *LUCAT1* knockout (*LUCAT1*-KO) and control (NC) groups in HCT116 and SW620 cells. GAPDH was used as an internal control. (**B**) Reduction in the proliferation ability of *LUCAT1*-KO HCT116 and SW620 cells compared with the control (NC) cells by a CCK8 assay. (**C**) Reduction in colony formation ability of *LUCAT1*-KO HCT116 and SW620 cells compared with that of the control (NC) cells by a colony formation assay. The bar graph indicates the number of colonies, *p < 0.05. (**D**) Cell cycle of *LUCAT1*-KO HCT116 and SW620 cells compared with the control (NC) was analyzed by flow cytometry. The distribution of the cell cycle is shown in the graphs. The results are presented as the mean±s.d. and are representative of at least three independent experiments. (**E**) Images of xenograft-transplantednude mousemodels (*n* = 6) and dissected tumors 30 days after injection with *LUCAT1*-KO SW620 cells, *LUCAT1*-KO HCT116 cells and their corresponding NC cell lines. (**F**) Tumor growth curves of *LUCAT1*-KO HCT116 and SW620 cell and control (NC) cell groups in the xenograft model. (**G**) Xenograft tumor weight for *LUCAT1*-KO HCT116 and SW620 cell and control (NC) cell groups in the xenograft model. The results are presented as the mean±s.d. and are representative of at least three independent experiments. *p < 0.05, **p < 0.01, ***p < 0.001, ****p < 0.0001, ns *p* > 0.05.

To clarify the functional significance of *LUCAT1* in CRC cells, we generated *LUCAT1*-knockout (*LUCAT1*-KO) cell lines for the first time by using the CRISPR/Cas9 system. The *LUCAT1*-specific dual sgRNAs were designed to knock out the full length of the *LUCAT1* coding sequence, excluding the possible biological activity of different *LUCAT1* transcripts as well. Cas9 and sgRNA were delivered by lentiviruses into HCT116 and SW620 cells. The deletion of *LUCAT1* was verified by genomic PCR using primers outside of the sgRNA-targeted region (positive control primers) and primers across the sgRNA-targeted region (negative control primers) (Supplementary Fig. 1a). The band detected in *LUCAT1*-KO and scramble cell lines (termed NC cells in the following text) confirmed the successful knockout of *LUCAT1*, and their sequences also correctly matched as expected (Supplementary Fig. 1b). Consistently, the mRNA levels of *LUCAT1* also dramatically decreased (Fig. 2A). Therefore, we investigated the role of *LUCAT1* in the malignant behavior of HCT116 and SW620 cells. Growth curves generated from CCK8 proliferation assays showed that *LUCAT1* knockout (*LUCAT1*-KO) significantly inhibited HCT116 and SW620 cell proliferation (Fig. 2B). Similarly, colony formation assays revealed that the clonogenic survival of HCT116 and SW620 cells was significantly impaired in *LUCAT1*-KO cell lines (Fig. 2C). However, our results showed that *LUCAT1* knockout did not induce cell cycle arrest (Fig. 2D). These findings suggested that *LUCAT1* played an important role in CRC proliferation in vitro.

To further evaluate the role of *LUCAT1* in vivo, we injected HCT116 *LUCAT1*-KO cells, SW620 *LUCAT1*-KO cells, and their corresponding NC cells into nude mice. All mice developed tumors at the injection site (Fig. 2E). However, the average size and weight of the tumors generated by *LUCAT1*-KO cells were significantly smaller than those generated by control cells (Fig. 2F, G). Consistently, immunohistochemical (IHC) staining showed a lower Ki67 expression in *LUCAT1*-KO tumor tissue compared to NC group (Supplementary Fig. 2a), suggesting a hindered proliferative ability of *LUCAT1*-KO cancer cells. All tumor tissues were verified by H&E staining (data not shown). Collectively, these results confirmed the oncogenic activity of *LUCAT1* in CRC in vivo, which was consistent with what we observed in vitro.

### *LUCAT1* directly binds to NCL in the nucleus

**Fig. 3:**
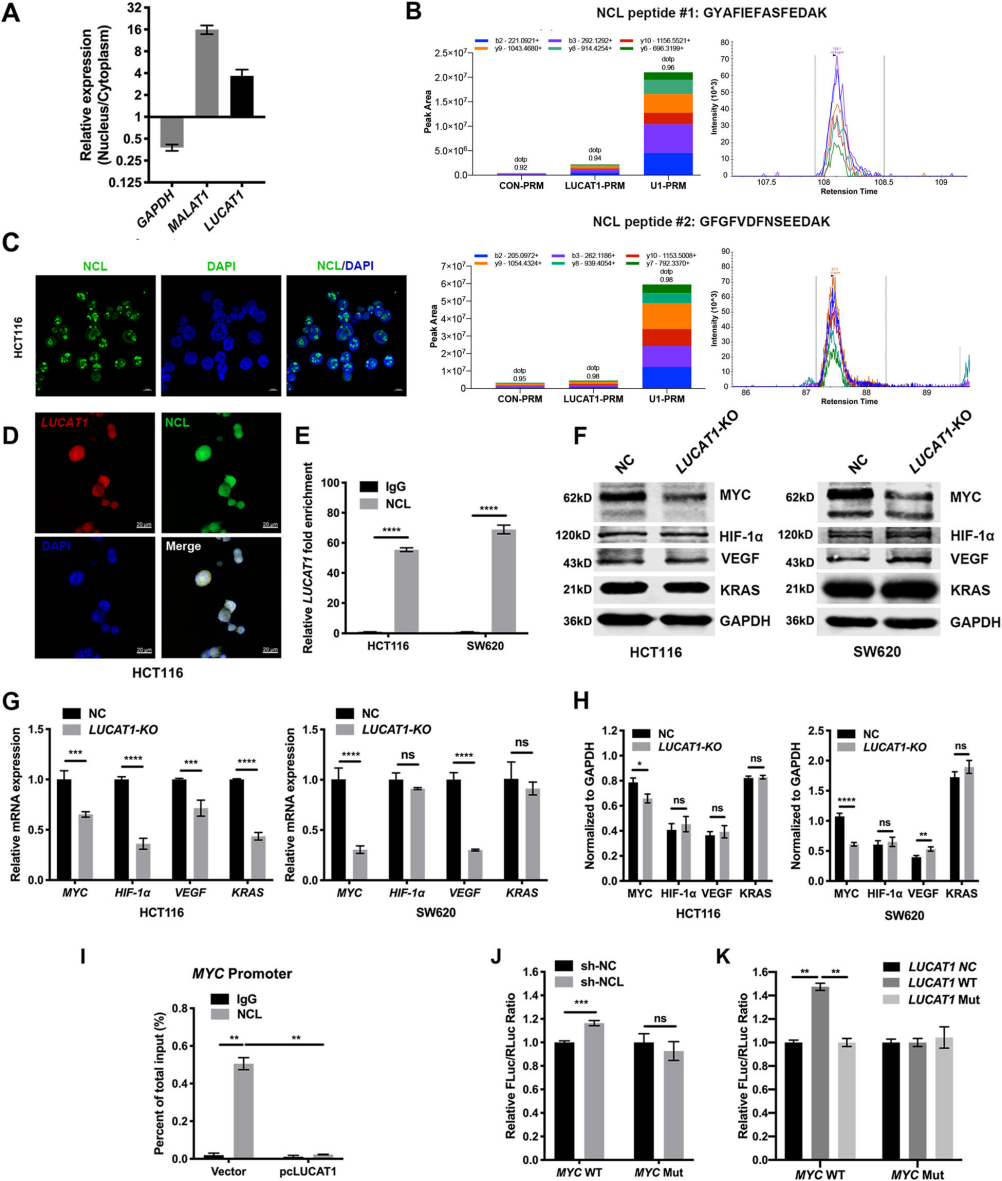
*LUCAT1* regulates MYC expression by binding to NCL in the nucleus. (**A**) The amounts of *LUCAT1* in the nuclear and cytoplasmic fractions of HCT116 cells were quantified by qRT-PCR. GAPDH was used as a control for cytoplasmic transcripts. MALAT1 was used as a nuclear marker. A nuclear/cytoplasmic ratio >1 proved that *LUCAT1* was a nuclear-enriched lncRNA. (**B**) ChIRP assay using *LUCAT1* probes followed by PRM analysis, which detected and quantified the amounts of NCL. Production pattern and chromatograph of two labeled NCL peptides (GYAFIEFASFEDAK and GFGFVDFNSEEDAK). Different colors represent different fragment ions of the same polypeptide. Each peptide was quantified using six fragment ions. U1 probes were used as a positive control. (**C**) Immunofluorescence staining using anti-NCL antibodies (green) in HCT116 cells. Scale bars, 10 µm. DAPI, 4′,6-diamidino-2-phenylindole. (**D**) Colocalization analysis: RNA FISH assay of *LUCAT1* (red) combined with the immunofluorescence detection of NCL (green) in HCT116 cells. Scale bars, 20 µm. (**E**) A RIP assay was performed in HCT116 and SW620 cells using anti-NCL and anti-IgG antibodies (control). The amount of *LUCAT1* mRNA was quantified by qRT-PCR. The results are presented as the mean±s.d. and are representative of at least three independent experiments. (**F**–**H**) Expression changes in four G4-associated genes, *MYC*, *HIF-1α*, *VEGF*, and *KRAS*, were determined in the NC and sgRNA HCT116 and SW620 cells with qRT-PCR (**G**) and Western blot analyses (**F**, **H**). (**I**) ChIP analysis of the interaction between the NCL protein and the *MYC* promoter in HCT116 cells. ChIP-qPCR of NCL at the *MYC* promoter when *LUCAT1* or the vector was overexpressed in HCT116 cells. (**J**) NCL binding to the *MYC* promoter was detected by a luciferase assay. The relative luciferase activity of the reporter containing the *MYC* NHE III_1_ promoter/mutant was cotransfected with the indicated constructs in HCT116 cells. *LUCAT1* interfered with NCL binding to the *MYC* promoter via its potential G4-forming sequence. (**K**) Relative luciferase activity of the reporter containing the *MYC* NHE III_1_ promoter/mutant that was cotransfected with the indicated constructs into HCT116 cells. The results are presented as the mean±s.d. and are representative of at least three independent experiments. **p < 0.01, ***p < 0.001, ****p < 0.0001, ns *p* > 0.05.

It has been shown that the function of lncRNAs is consistent with their subcellular localization and exerted by interacting with various proteins^30^. We first performed assays in HCT116 cells to separate the cytoplasmic and nuclear RNA components and measured the expression ratio of *LUCAT1* by qRT-PCR to confirm the subcellular localization of *LUCAT1*. We found that *LUCAT1* was predominantly enriched in the nuclei of CRC cells (Fig. 3A). One of the most important roles of lncRNAs in the nucleus is their interaction with specific proteins to regulate the expression of target genes^5,31–33^; thus, we speculated that *LUCAT1* may also function in this manner. To verify our hypothesis, we used biotinylated *LUCAT1* probes to investigate proteins that potentially interact with *LUCAT1* by performing ChIRP assays coupled with mass spectrometry (LC–MS/MS) in HCT116 cells and SW620 cells. In total, 52 specific *LUCAT1*-associated proteins were isolated from HCT116 cells, 17 specific *LUCAT1*-associated proteins were isolated from SW620 cells, and only NCL, RPL18A, and C7orf24 were detected in both cells (Supplementary Table 6). Among these proteins, NCL was selected for further verification due to its better mass spectrometric data and a higher binding score (Supplementary Table 7). Then, the parallel reaction monitoring (PRM) results further verified and quantified NCL among the products precipitated by the *LUCAT1* probes, showing that compared with the negative control, the product was enriched with NCL, indicating that NCL might be the *LUCAT1* interaction protein (Fig. 3B). To validate the potential interaction between *LUCAT1* and NCL, we performed RIP assays in HCT116 and SW620 cells. The results revealed that *LUCAT1* was significantly more enriched with the anti-NCL antibody than with the IgG (control antibody) (Fig. 3E). Furthermore, we simultaneously carried out FISH with the *LUCAT1* probe and IF with the anti-NCL antibody. The results showed that NCL was significantly abundant in the nucleus (Fig. 3C), and the colocalization of *LUCAT1* and NCL in the nucleus was clearly observed in HCT116 cells (Fig. 3D). Taken together, these findings demonstrated that *LUCAT1* is an NCL-binding lncRNA.

### *LUCAT1* regulates MYC expression by antagonizing the function of NCL

It has been demonstrated that NCL can regulate gene expression by binding to the G4 sequence and promoting G4 structure formation with its high affinity and selectivity^18,34^. Therefore, we hypothesized that *LUCAT1* has a potential G4 sequence that can be recognized by NCL and then affected NCL-mediated regulation of gene expression. To verify this idea, we first screened several important oncogenes, including KRAS, MYC, VEGF, and HIF-1α, by qPCR and Western blot analyses; these oncogenes are reported to contain the G4 structure in their promoters and are closely associated with CRC^34–37^. We found that *MYC* mRNA and protein expression in *LUCAT1*-KO HCT116 and *LUCAT1*-KO SW620 cells was significantly decreased compared with that in NC cells (Fig. 3F–H). Down-regulation of MYC was also observed in *LUCAT1*-KO tumor tissue by IHC staining than NC group (Supplementary Fig. 2b). Further bioinformatic analysis revealed that *LUCAT1* was correlated with *MYC* expression in CRC cancers (Supplementary Fig. 3). Together, these data suggest that MYC transcription is regulated by *LUCAT1* in NCL-dependent way.

To determine whether NCL controls the transcription of *MYC* through the binding of G4-forming sequence in the promoter region, we next performed a ChIP assay in HCT116 cells. The ChIP results confirmed that NCL could directly bind to the G4 sequence of *MYC* (-141 bp to -114 bp) in the promoter region (named *MYC* NHE III_1_^38^) (Supplementary Fig. 4b), while fragments of *KRAS*, *VEGF* and *HIF-1α* were not observed (Supplementary Fig. 4a).

To further confirm that NCL could directly bind to *MYC* NHE III_1_ and regulate gene expression, we assayed the expression of a luciferase reporter containing *MYC* NHE III_1_ sequences (-1000 bp to 0 bp). Because HCT116 cells expressed a high basal level of NCL, we first knocked down NCL in HCT116 cells by using NCL-specific small hairpin RNAs (sh-NCL) (Supplementary Fig. 5). We found that inhibition of NCL resulted in increased reporter luciferase activity, but this increase was abrogated when the G4 sequence of *MYC* NHE III_1_ was mutated (Fig. 3J).

Due to the high affinity of NCL for G4, we analyzed the potential G4 sequence of *LUCAT1* using the QGRS Mapper program. The QGRS Mapper program predicted that the putative G4-forming region from nucleotides 717 to 746 of *LUCAT1* (*LUCAT1* 717-746) had the highest score for G4-forming probability (Supplementary Table 8). We next investigated whether *LUCAT1* 717-746 was required for its interaction with NCL. We introduced mutations into the *LUCAT1* region 717–746 to generate *LUCAT1*-Mut. We found that overexpression of *LUCAT1* resulted in increased *MYC* reporter luciferase activity but not *LUCAT1*-Mut luciferase activity (Fig. 3K), indicating that the NCL-mediated inhibition of *MYC* transcription could be reduced by overexpressing *LUCAT1*. In addition, overexpression of both *LUCAT1* and *LUCAT1*-Mut did not change the reporter luciferase activity when the G4 sequence of *MYC* NHE III_1_ was mutated (Fig. 3K). Consistently, overexpression of *LUCAT1* also resulted in decreased binding affinity between NCL and the *MYC* promoter in the ChIP assay (Fig. 3I). These findings suggested that *LUCAT1* may directly interact with NCL through the *LUCAT1* region 717–746.

We further compared the sequence of *LUCAT1* 717-746 (Supplementary Fig. 4c, highlighted) with the sequence of PCR products produced by the RIP assay with an anti-NCL antibody (Supplementary Fig. 4c, underlined); interestingly, there was an overlap between them. In other words, these findings further verified the accuracy and correctness of the prediction of the binding site between *LUCAT1* and NCL. Taken together, these results indicated that the G4-forming sequence of *LUCAT1* could antagonize NCL binding to *MYC* NHE III_1_ in the promoter.

### Silencing NCL restores *LUCAT1*-KO-mediated inhibition of MYC expression and CRC cell proliferation

**Fig. 4:**
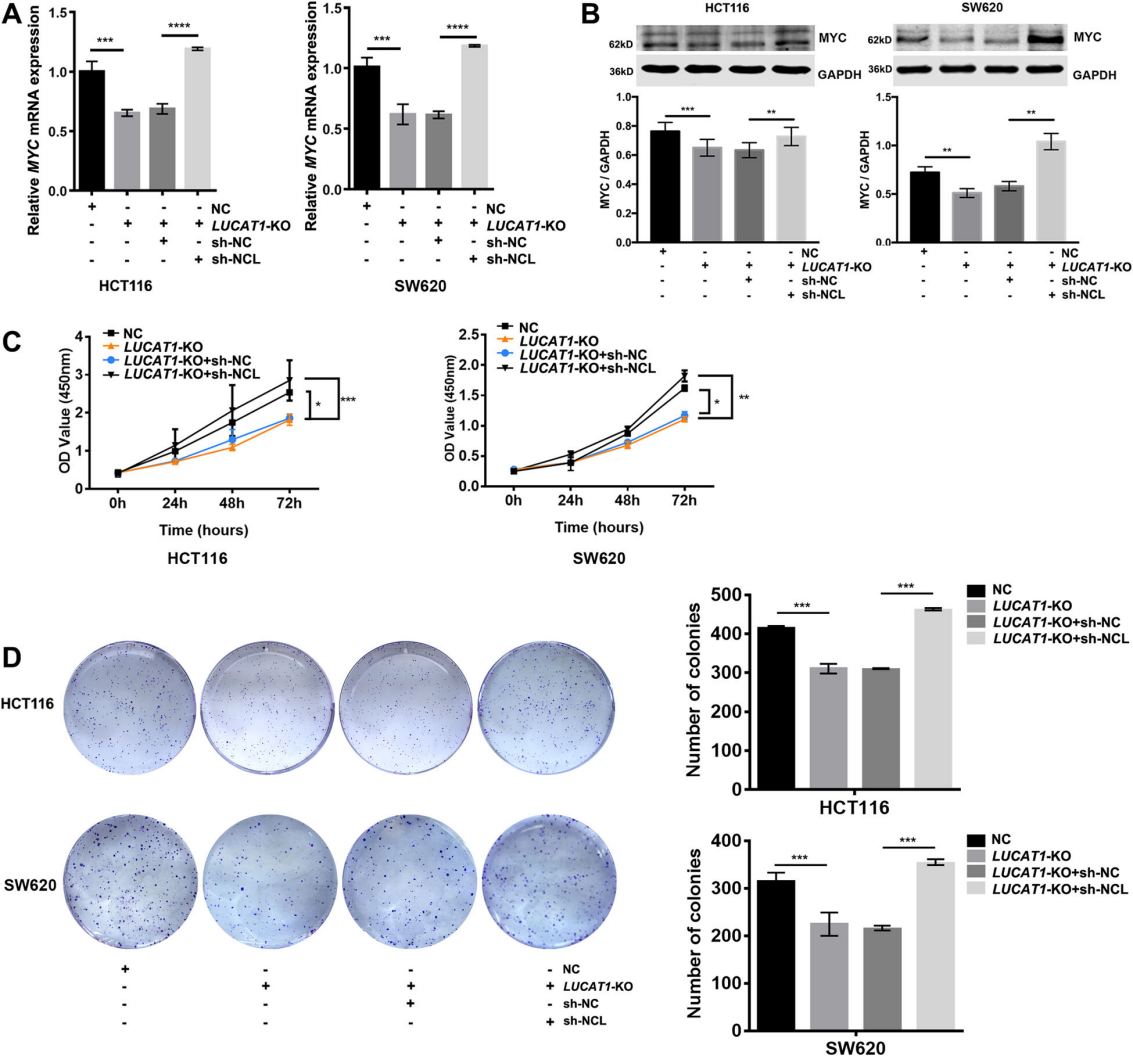
Knockdown of NCL restores MYC expression and cell proliferation in *LUCAT1*-knockout CRC cells. (**A**) *LUCAT1*-KO HCT116 and SW620 cells were transfected with the indicated shRNAs, and *MYC* expression was quantified by qRT-PCR. GAPDH was used as a control. (**B**) *LUCAT1*-KO HCT116 and SW620 cells were transfected with the indicated shRNAs, and MYC expression was quantified by a Western blot. GAPDH was used as a control. (**C**) *LUCAT1*-KO HCT116 and SW620 cells were transfected with the indicated shRNAs, and the proliferation ability was quantified by a CCK8 assay. (**D**) *LUCAT1*-KO HCT116 and SW620 cells were transfected with the indicated shRNAs, and the colony formation ability was quantified by a colony formation assay. The results are presented as the mean ± s.d. and are representative of at least three independent experiments. *p < 0.05, **p < 0.01, ***p < 0.001, ****p < 0.0001.

To investigate the function of NCL in the *LUCAT1*-mediated modulation of MYC expression and proliferation, we examined the effect of NCL knockdown on MYC expression, as well as on the proliferation activity of *LUCAT1*-KO HCT116 and SW620 cells. We found that knockdown of NCL almost completely restored MYC expression in *LUCAT1*-KO HCT116 and SW620 cells (Fig. 4A, B). In addition, knockdown of NCL also restored the proliferative activities of *LUCAT1*-KO HCT116 and SW620 cells (Fig. 4C, D). These results further indicated that *LUCAT1* promoted CRC cell proliferation by antagonizing the NCL-mediated inhibitory effects on MYC.

## Discussion

Recent studies have shown that thousands of lncRNAs are dysregulated in many types of cancer and play important roles in carcinogenesis, but few studies have elucidated their specific mechanisms. In this study, we discovered that *LUCAT1* promoted CRC cell proliferation by forming a G4 with NCL. This interaction blocked NCL from facilitating G4 formation in the *MYC* gene promoter region and led to MYC expression.

Our present study demonstrates the clinical significance of *LUCAT1* in CRC. The clinical data revealed that *LUCAT1* in CRC tissues was upregulated compared with that in adjacent normal tissues, indicating that *LUCAT1* may be involved in the development and progression of CRC. We further investigated the relationship between *LUCAT1* and clinical pathology of CRC and verified that higher *LUCAT1* expression resulted in poorer histopathological grade and increased lymph node metastasis than lower *LUCAT1* expression. Chen et al’s study shows that *LUCAT1* up-regulation is significantly associated with CRC liver metastasis and poorer clinical prognosis^18^, supporting our findings that *LUCAT1* is contributed to progression of CRC. In addition, a systematic analysis of online databases in our study discovered that *LUCAT1* could be a risk factor for prognosis in CRC patients, suggesting it might be a potential therapeutic target for CRC. Similar condition is reported in cervical cancer^39^ and non-small-cell lung cancer^15^, showing that highly expressed *LUCAT1* in peripheral blood or tissue of patients is related to poorer survival. To further investigate the oncogenic role of *LUCAT1* in CRC, we transfected the CRISPR/Cas9 system with dual sgRNAs into HCT116 and SW620 cells to knock out the full-length *LUCAT1* gene coding sequence. Compared with other studies, our study showed a causal role of *LUCAT1* in cancer via the CRISPR/Cas9 system for the first time. Although shRNA offers the ability to create cells with stable suppression of individual genes, a genome-wide collection of individual gene-silenced cells is currently unavailable for any mammal. Conversely, CRISPR/Cas9-based gene-deleted cells are available for any mammal^40^. After knocking out *LUCAT1*, CRC cell proliferation was significantly inhibited. This is also consistent with the results of studies in cervical cancer, ovarian cancer, breast cancer, choroidal melanoma, clear cell renal cell carcinoma, non-small-cell lung cancer and esophageal squamous cell carcinoma, showing that loss of *LUCAT1* hinders cell proliferation^15,17,20,39,41–43^. The results described above indicate the oncogenic role of *LUCAT1* in CRC.

Like other lncRNAs, *LUCAT1* has been proven to participate in carcinogenesis and cancer progression by regulating gene expression through a posttranscriptional mechanism^19^. For example, *LUCAT1* can promote esophageal squamous cell carcinoma and clear cell renal cell carcinoma tumorigenesis by controlling the ubiquitination and stability of DNMT1 and the AKT/GSK-3β signaling pathway, respectively^17,20^. In addition, *LUCAT1* has been reported to induce CRC cell cycle arrest and apoptosis by binding to UBA52 and activating the p53 pathway^44^. Some studies also explore the relationship between *LUCAT1* and miRNAs, including miR-514a/b-3b in choroidal melanoma, miR-199a-5p in ovarian cancer, and miR-199b-5p in cervical cancer^39,41,42^. *LUCAT1* are found to share conserved binding site with these miRNAs and then modulates cancer progression through their direct interaction. However, the mechanisms of *LUCAT1* transcriptional regulation in CRC remain unclear. To further investigate the underlying mechanisms by which *LUCAT1* regulated downstream effectors in CRC, ChIRP and RIP experiments were performed. Proteins interacting with *LUCAT1* in CRC were pulled down by biotin-labeled *LUCAT1* probes and investigated by LC/MS analysis, and NCL was identified as a *LUCAT1*-associated protein for the first time. *LUCAT1* also could be precipitated by NCL antibody, confirming that *LUCAT1* were directly interacted with NCL in CRC cells. We demonstrated that the depletion of NCL restored the cell proliferation ability of CRC cells with *LUCAT1* knockout. These results indicate that *LUCAT1* enhances CRC cell proliferation by negatively regulating NCL function.

It has been demonstrated that NCL localizes primarily in the nucleus and binds to the G4 to facilitate its formation^38,45,46^. The presence of G4s can be found in the promoter regions of many oncogenes. It has been reported that NCL binds to the G4 in the promoter, and this binding suppresses oncogene expression^34^. Some small RNAs have also been reported to stabilize or interfere with the G4 structure^24,47^. Therefore, we investigated four candidate genes, *MYC*, *KRAS*, *VEGF*, and *HIF-1α*, which were dysregulated in CRC and closely correlated with CRC progression^48–51^. They are also known to form G4 structures in their promoter regions and negatively regulate gene transcription^22,34,36,52^. We found that only MYC could be suppressed when *LUCAT1* was knocked out, and this reduction could be reversed by knocking down NCL. Then, both ChIP and luciferase reporter assays confirmed that NCL could directly bind to *MYC* NHE III_1_, the promoter region that controlled up to 90% of total *MYC* transcription. Overexpression of *LUCAT1* blocked NCL from binding to the *MYC* promoter and enhanced *MYC* transcription. Our results support the hypothesis that NCL can regulate *MYC* transcription in HeLa cells^38^. Thus, we disclosed that *LUCAT1* enhanced *MYC* transcription and expression by interacting with NCL, which prevented NCL from being recruited to the *MYC* promoter and inducing G4 formation. Although other proteins, such as UBA52, were reported to interact with *LUCAT1* in HCT116 cells^53^, they were not detected in our ChIRP-MS results. A possible reason is that *LUCAT1* is multifunctional and can regulate CRC progression through a different pathway.

Some studies have demonstrated that lncRNAs can act as molecular decoys to competitively bind to proteins by imitating their G4-forming structure sequence^23^. Since NCL can target G4 and facilitate its formation, we speculated that NCL might also be able to bind to *LUCAT1* via putative G4-forming regions in *LUCAT1*. As we expected, the *MYC* reporter luciferase activity inhibited by NCL could be recovered by the overexpression of *LUCAT1* but not by *LUCAT1*-Mut, which was mutated at the sequence of the predicted G4-forming region. Moreover, the sequence of the predicted G4-forming region in *LUCAT1* was consistent with the sequences of the RNA fragments detected in the RIP experiment. These results indicate that nucleotides 716 bp to 746 bp of the *LUCAT1* transcript contain a G4-forming sequence, which could be recognized by NCL to form G4 structures.

In summary, our present work revealed that the CRC-associated lncRNA *LUCAT1* played an oncogenic role, contributing to CRC proliferation. We confirmed for the first time that *LUCAT1* interfered with G4 formation in the *MYC* promoter by antagonizing the binding between NCL and MYC via its G4-forming region, promoting *MYC* transcription and expression. In conclusion, these results might indicate that the *LUCAT1*/NCL/MYC axis might be a potential prognostic marker and therapeutic target in CRC, potentially providing new insight into CRC progression and treatment.

## Supplementary information

Supplementary Figure4

Supplementary Figure5

Supplementary Figure Legend

Supplementary Figure1

Supplementary Figure2

Supplementary Figure3

Supplementary Table1

Supplementary Table2

Supplementary Table3

Supplementary Table4

Supplementary Table5

Supplementary Table6

Supplementary Table7

Supplementary Table8

Supplementary Table9

## Data Availability

Gene expression analysis of *LUCAT1* was carried out using the starBase database v3.0 project (http://starbase.sysu.edu.cn). The survival analysis of *LUCAT1* was carried out using the Gene Expression Profiling Interaction Analysis database (http://gepia.cancer-pku.cn). The relationship between *LUCAT1* and NCL was predicted via RNA-Protein Interaction Prediction (http://pridb.gdcb.iastate.edu/RPISeq/). The potential binding sites between *LUCAT1* and NCL were predicted by QGRS Mapper (http://bioinformatics.ramapo.edu/QGRS/index.php). All the data will be made available upon reasonable request.
