# Supplementary figures and images for: The long noncoding RNA LUCAT1 promotes colorectal cancer cell proliferation by antagonizing Nucleolin to regulate MYC expression
Source: Cell Death Dis. 2020 Oct 23;11(10):908. doi: 10.1038/s41419-020-03095-4 (PMC7584667; doi:10.1038/s41419-020-03095-4)

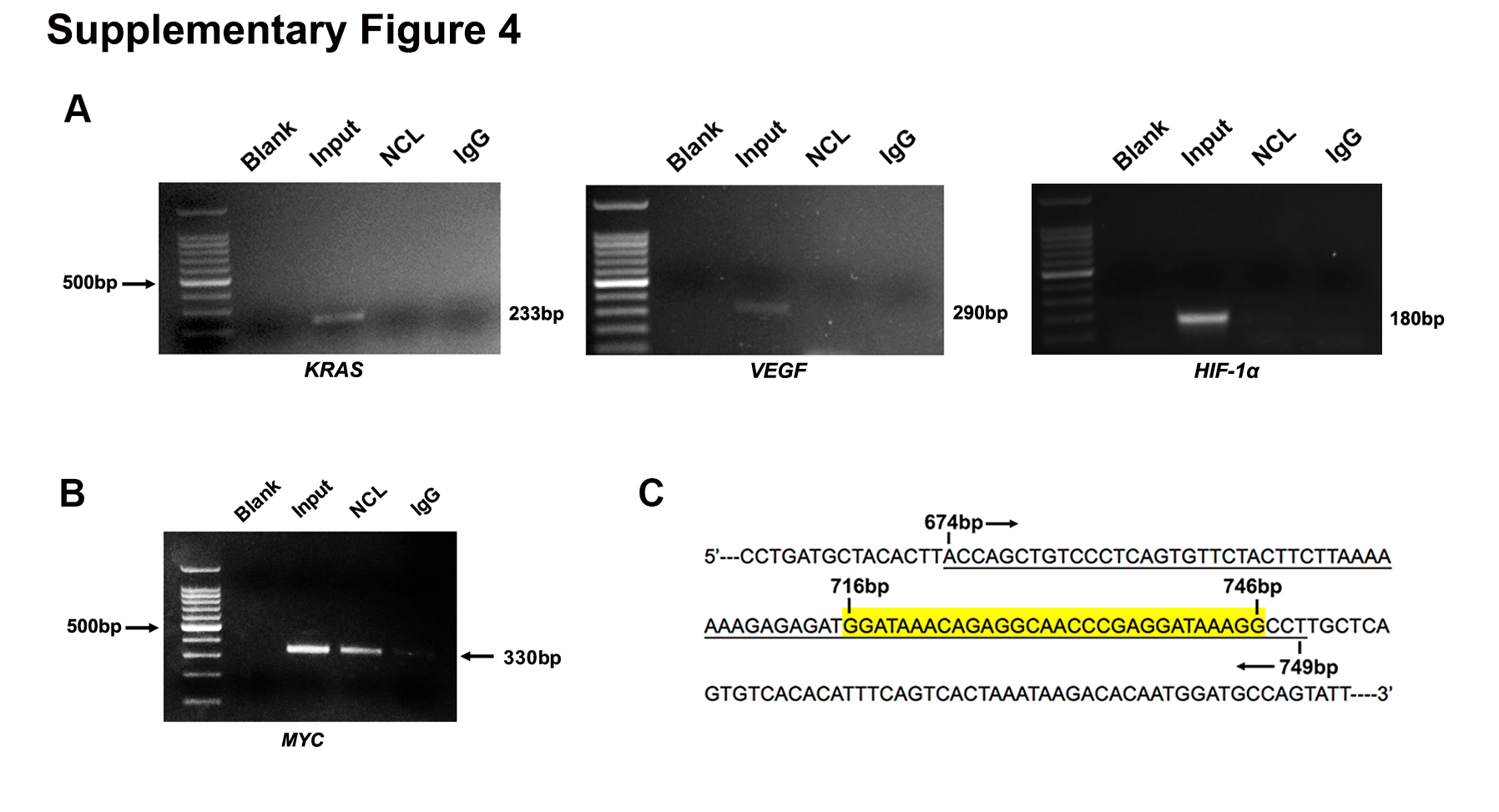

Supplement: Supplementary file 1 — Supplementary Figure4 [file 41419_2020_3095_MOESM1_ESM.tif]

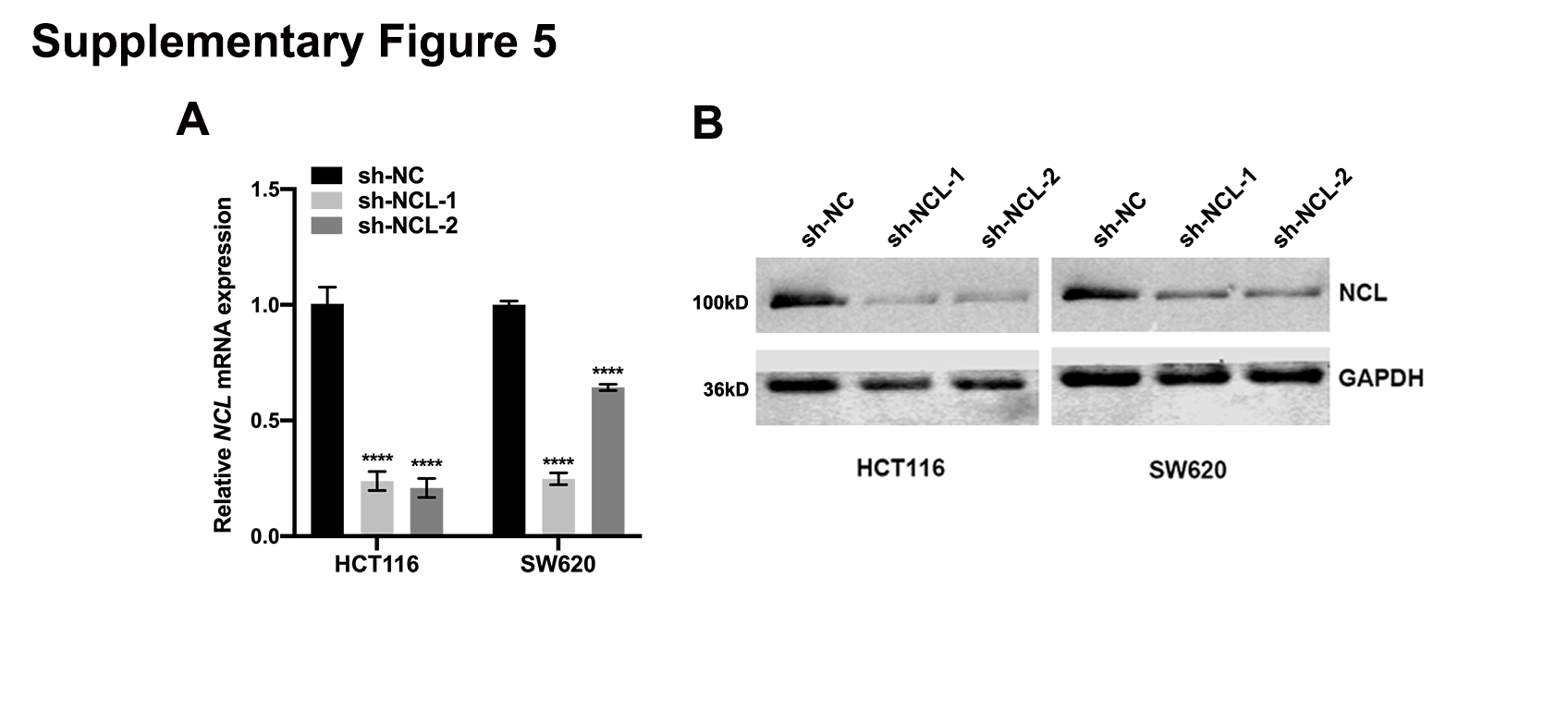

Supplement: Supplementary file 2 — Supplementary Figure5 [file 41419_2020_3095_MOESM2_ESM.tif]

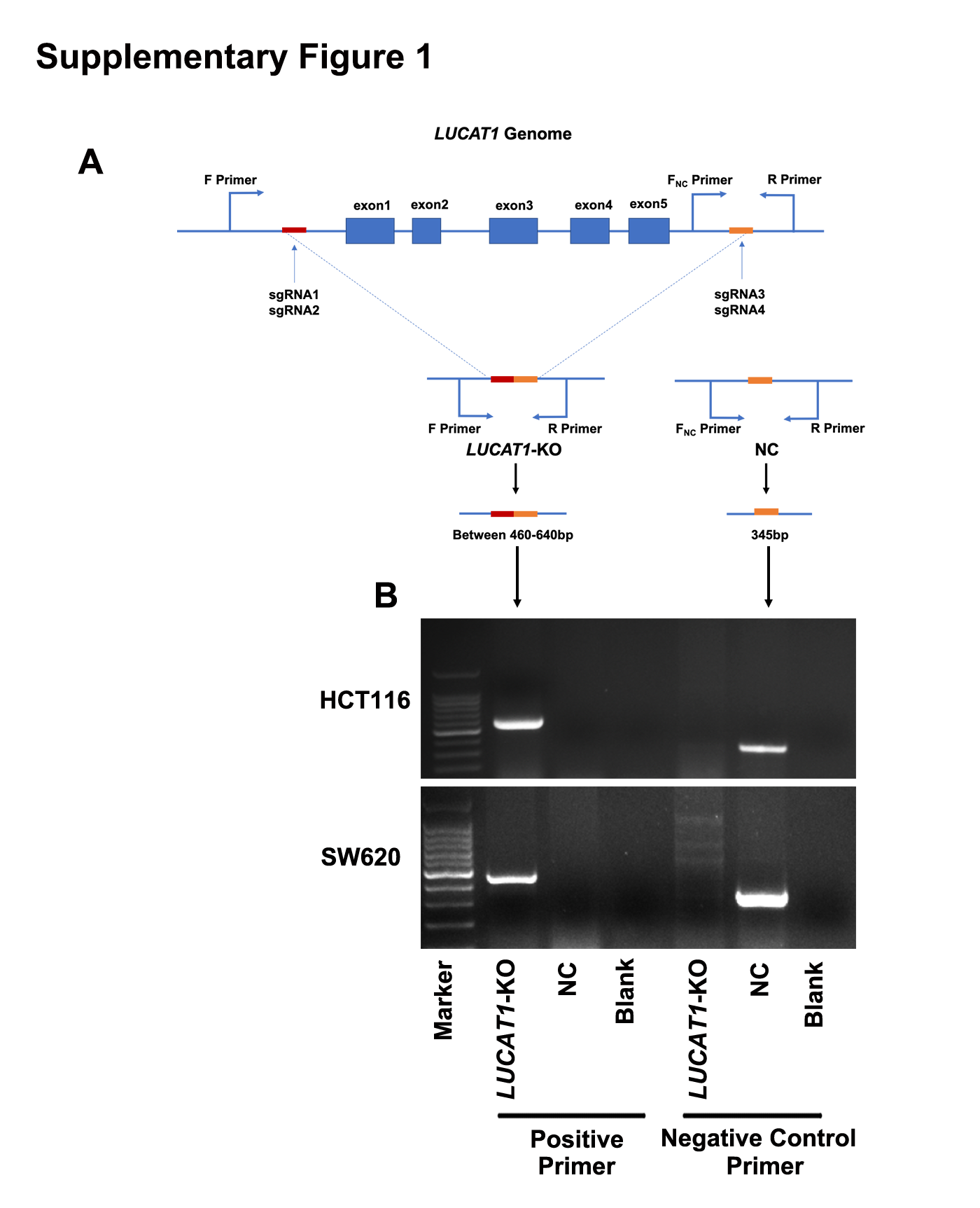

Supplement: Supplementary file 4 — Supplementary Figure1 [file 41419_2020_3095_MOESM4_ESM.tif]

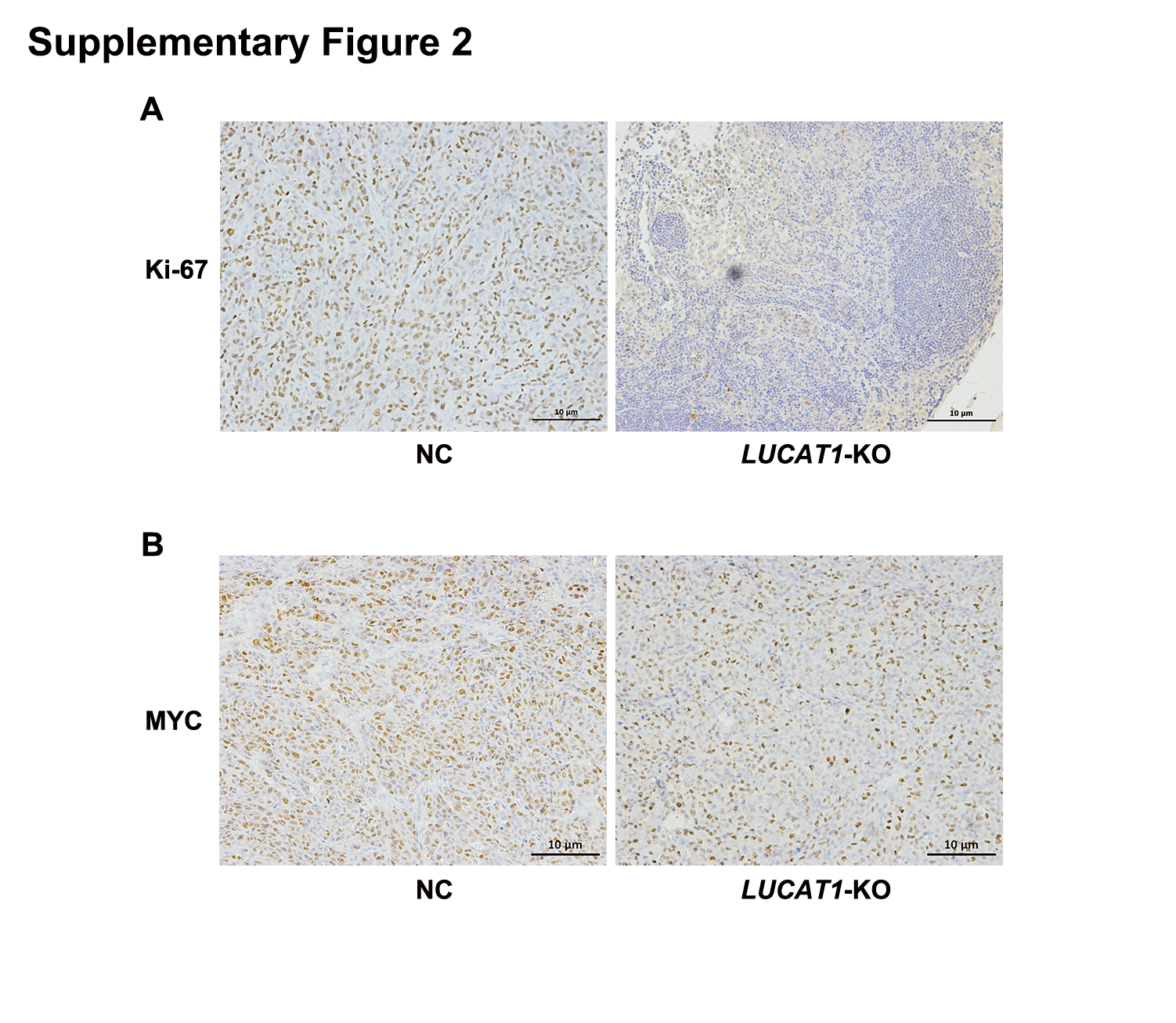

Supplement: Supplementary file 5 — Supplementary Figure2 [file 41419_2020_3095_MOESM5_ESM.tif]

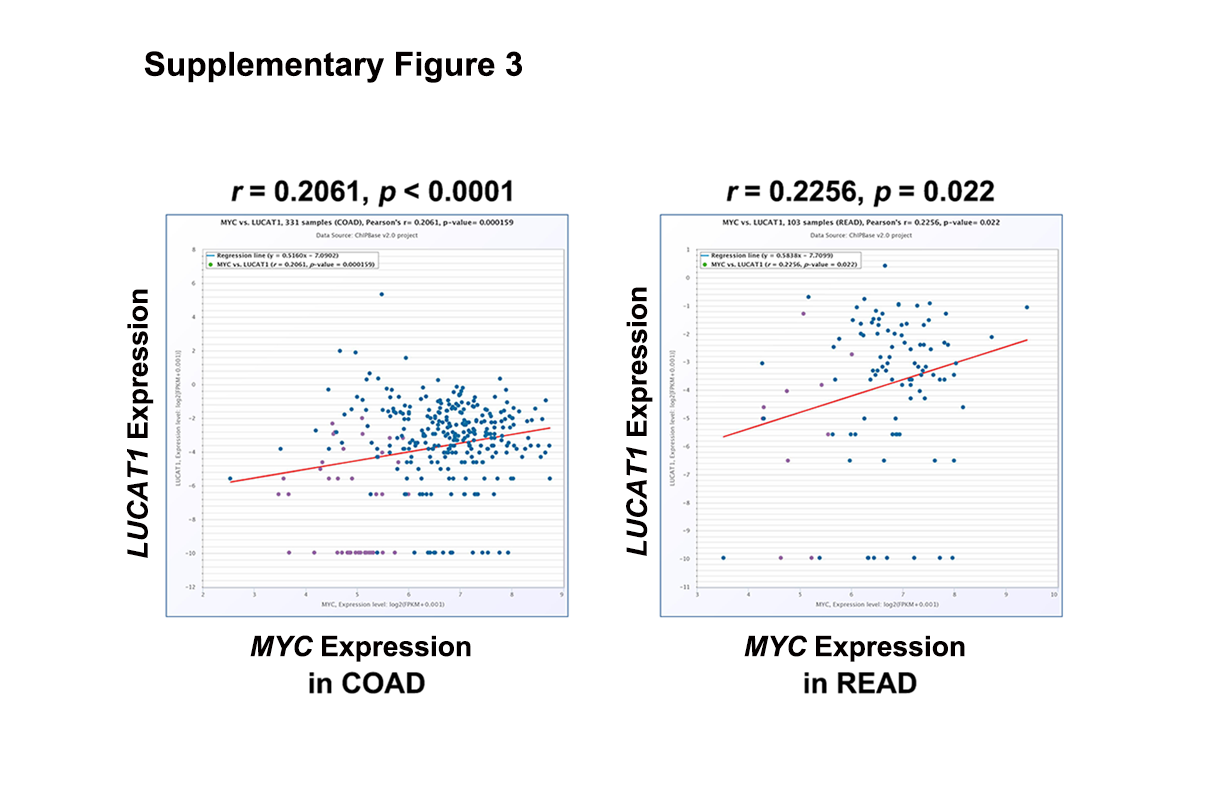

Supplement: Supplementary file 6 — Supplementary Figure3 [file 41419_2020_3095_MOESM6_ESM.tif]
