# Supplementary material for: The long noncoding RNA LUCAT1 promotes colorectal cancer cell proliferation by antagonizing Nucleolin to regulate MYC expression
Source: Cell Death Dis. 2020 Oct 23;11(10):908. doi: 10.1038/s41419-020-03095-4 (PMC7584667; doi:10.1038/s41419-020-03095-4)
Supplement: Supplementary file 3 — Supplementary Figure Legend [file 41419_2020_3095_MOESM3_ESM.pdf]

Supplementary Figure 1: Knockout of *LUCAT1* by CRISPR/Cas9 system in the HCT116 and SW620 cells.

(**A**) Schematic diagram of human *LUCAT1* gene and sgRNA target-site position. Four target sites were designed based on the full-length *LUCAT1* gene-coding sequence, and two pairs of special primers were design to confirm knockout efficiency base on the sgRNA target sites. F primer: positive forward primer; R primer: positive reverse primer/negative control reverse primer; F_{NC} primer: negative control forward primer. (**B**) PCR products of targeted region of *LUCAT1* in HCT116 and SW620 cells. Amplification of DNA fragments using positive primers (line 1) showed homozygous mutation with a full-length *LUCAT1* gene-coding sequence deletion.

Supplementary Figure 2: IHC staining of xenograft tumors.

Tissue paraffin sections from representative tumors were used for immunohistochemical staining for Ki67 (**A**) and MYC (**B**).

Supplementary Figure 3: Positive correlation between *LUCAT1* and *MYC*.

Correlation analysis of *LUCAT1* and *MYC* expression in COAD and READ based on TCGA database. COAD: Colon adenocarcinoma; READ: Rectum adenocarcinoma.

Supplementary Figure 4: NCL binds to G4 sequence.

Amplification of putative G4 sequence in *HIF-1α*, *VEGF* and *KRAS* promoter region (**A**) or *MYC* NHE III_1 region (**B**) pulled down by anti-NCL antibody from ChIP assay. (**C**) Predicted G4-forming region sequence of *LUCAT1* (**highlighted**) and sequence of *LUCAT1* detected by RIP assay with an anti-NCL antibody (**underlined**).

Supplementary Figure 5: Knockdown of NCL by shRNA in the HCT116 and SW620 cells.

qRT-PCR (**A**) and Western blot analysis(**B**) of NCL expression in sh-NC, sh-NCL-1 and sh-NCL-2. GAPDH was used as an internal control. The results are presented as the mean±s.d. and are representative of at least three independent experiments. ****$p<0.0001$.
