# Supplementary material for: The long noncoding RNA LUCAT1 promotes colorectal cancer cell proliferation by antagonizing Nucleolin to regulate MYC expression
Source: Cell Death Dis. 2020 Oct 23;11(10):908. doi: 10.1038/s41419-020-03095-4 (PMC7584667; doi:10.1038/s41419-020-03095-4)
Supplement: Supplementary file 7 — Supplementary Table1 [file 41419_2020_3095_MOESM7_ESM.doc]

**Supplementary Table 1. Sequences of sgRNA and shRNA**

|  |  |
| --- | --- |
| Name | Sequence 5’---3’ |
| *LUCAT1* sgRNA1 | GTCTGTAATCCCCGGTACTC |
| *LUCAT1* sgRNA2 | CTCAGTAACAAAATCTAGGC |
| *LUCAT1* sgRNA3 | CTAGTTGGCGATGCATCCAC |
| *LUCAT1* sgRNA4 | GTAAAGGCCGCTGGAACTGA |
| sh-NCL-1 | CCGGCGGTGAAATTGATGGAAATAACTCGAGTTATTTCCATCAATTTCACCGTTTTTG |
| sh-NCL-2 | CCGGAGTAAAGGGATTGCTTATATTCTCGAGAATATAAGCAATCCCTTTACTTTTTTG |
| sh-NC | CCGGTTCTCCGAACGTGTCACGTTTCAAGAGAACGTGACACGTTCGGAGAATTTTTG |
