# Supplementary material for: The long noncoding RNA LUCAT1 promotes colorectal cancer cell proliferation by antagonizing Nucleolin to regulate MYC expression
Source: Cell Death Dis. 2020 Oct 23;11(10):908. doi: 10.1038/s41419-020-03095-4 (PMC7584667; doi:10.1038/s41419-020-03095-4)
Supplement: Supplementary file 8 — Supplementary Table2 [file 41419_2020_3095_MOESM8_ESM.doc]

**Supplementary Table 2. qRT-PCR and PCR** primer sequences

| Primer name | Sequence 5’---3’ |
| --- | --- |
| *LUCAT1* FW | GCTCGGATTGCCTTAGACAG |
| *LUCAT1* RW | GGGTGAGCTTCTTGTGAGGA |
| *NCL* FW | GCGTTGGAACTCACTGGTTT |
| *NCL* RW | CCGCAGCATCTTCAAACACT |
| *MYC* FW | CACCAGCAGCGACTCTGA |
| *MYC* RW | GATCCAGACTCTGACCTTTTGC |
| *KRAS* FW | TGGACGAATATGATCCAACAAT |
| *KRAS* RW | TCCCTCATTGCACTGTACTCC |
| *HIF-1α* FW | ATGTAGTAGCTGCATGATCGTCT |
| *HIF-1α* RW | ATGTAGTAGCTGCATGATCGTCT |
| *VEGF* FW | TGAGTTAAACGAACGTACTTGCAG |
| *VEGF* RW | TGTATCGATCGTTCTGTATCAGTCT |
| *GAPDH* FW | TGCACCACCAACTGCTTAGC |
| *GAPDH* RW | GGCATGGACTGTGGTCATGAG |
| Positive Primer for *LUCAT1* in genome DNA FW | AGTCTTTGATATGGAGACAGACTAG |
| Positive Primer for *LUCAT1* in genome DNA RW | GAGCCACCATACCAAGCCTGTTTGC |
| Negative Control Primer for *LUCAT1* in genome DNA FW | TGAAATGTTCCAGCTTTGGG |
| Negative Control Primer for *LUCAT1* in genome DNA RW | GAGCCACCATACCAAGCCTGTTTGC |
