# Supplementary material for: The long noncoding RNA LUCAT1 promotes colorectal cancer cell proliferation by antagonizing Nucleolin to regulate MYC expression
Source: Cell Death Dis. 2020 Oct 23;11(10):908. doi: 10.1038/s41419-020-03095-4 (PMC7584667; doi:10.1038/s41419-020-03095-4)
Supplement: Supplementary file 9 — Supplementary Table3 [file 41419_2020_3095_MOESM9_ESM.doc]

**Supplementary Table 3. Sequences of ChIRP** biotin probes

| Probe number | Probe sequence 5’---3’  (Antisense of *LUCAT1*) | Probe locations (start) |
| --- | --- | --- |
| *LUCAT1* Probe 1 | GTCTGAGTGGAGTGTTGATT | 2 |
| *LUCAT1* Probe 2 | AGAGGATGAAAGCTGTTCTT | 81 |
| *LUCAT1* Probe 3 | ATAAGAGTTCCAGCTGGGTG | 161 |
| *LUCAT1* Probe 4 | CATGGTAGATGCTGAACCAA | 284 |
| *LUCAT1* Probe 5 | GACACAACTGTACAGGCACG | 566 |
| *LUCAT1* Probe 6 | GCATCCATTGTGTCTTATTT | 777 |
| *LUCAT1* Probe 7 | TGACTGCAAGAGCTTGAAGG | 827 |
