# Supplementary material for: The long noncoding RNA LUCAT1 promotes colorectal cancer cell proliferation by antagonizing Nucleolin to regulate MYC expression
Source: Cell Death Dis. 2020 Oct 23;11(10):908. doi: 10.1038/s41419-020-03095-4 (PMC7584667; doi:10.1038/s41419-020-03095-4)
Supplement: Supplementary file 10 — Supplementary Table4 [file 41419_2020_3095_MOESM10_ESM.doc]

**Supplementary Table 4. Primary and secondary antibodies for Western blot**

| Antibody | Host | Vendor |
| --- | --- | --- |
| anti-NCL | Rabbit | Cell Signaling Technology, #14574 |
| anti-MYC | Rabbit | Proteintech, #10828-1-AP |
| anti-KRAS | Rabbit | Proteintech, #12063-1-AP |
| anti-HIF-1α | Rabbit | Proteintech, #20960-1-AP |
| Anti-VEGF | Rabbit | Proteintech, #19003-1-AP |
| anti-GAPDH | Mouse | Proteintech, #60004-1-Ig |
| IRDye 800CW anti-Rabbit IgG | Goat | LI-COR Biosciences, 925-32210 |
| IRDye 800CW anti-Mouse IgG | Goat | LI-COR Biosciences, 925-32211 |
