# Supplementary material for: The long noncoding RNA LUCAT1 promotes colorectal cancer cell proliferation by antagonizing Nucleolin to regulate MYC expression
Source: Cell Death Dis. 2020 Oct 23;11(10):908. doi: 10.1038/s41419-020-03095-4 (PMC7584667; doi:10.1038/s41419-020-03095-4)
Supplement: Supplementary file 11 — Supplementary Table5 [file 41419_2020_3095_MOESM11_ESM.doc]

**Supplementary Table 5. ChIP** PCR primer sequences

| Primer name | Sequence 5’---3’ |
| --- | --- |
| *MYC* NHE III FW | AGTCTTTGATATGGAGACAGACTAG |
| *MYC* NHE III RW | GAGCCACCATACCAAGCCTGTTTGC |
| *KRAS* NHE III FW | TTGCCTTGCTCACCATTA |
| *KRAS* NHE III RW | TCCTCTGTCCGTCTACTG |
| *HIF-1α* NHE III FW | TGTGCACTGAGGAGCTGAG |
| *HIF-1α* NHE III RW | GCCCGACACACTGGCCGAAGCGACGA |
| *VEGF* NHE III FW | CGGGGGCGGATGGGTAAT |
| *VEGF* NHE III RW | CCCAGCGCCACGACCTCCGA |
