# Supplementary material for: The long noncoding RNA LUCAT1 promotes colorectal cancer cell proliferation by antagonizing Nucleolin to regulate MYC expression
Source: Cell Death Dis. 2020 Oct 23;11(10):908. doi: 10.1038/s41419-020-03095-4 (PMC7584667; doi:10.1038/s41419-020-03095-4)
Supplement: Supplementary file 13 — Supplementary Table7 [file 41419_2020_3095_MOESM13_ESM.doc]

**Supplementary Table 7. Interaction possibility between proteins and *LUCAT1***

| Proteins &LUCAT1 | Prediction using RF classifier | Prediction using SVM classifier |
| --- | --- | --- |
| NCL | 0.7 | 0.85 |
| RPL18 | 0.65 | 0.86 |
| C7orf24 | 0.75 | 0.84 |
| Predictions with probabilities>0.5 were considered “positive”, indicating the corresponding RNA and protein are likely to interact. RF refers to random forest. SVM refers to support vector machine. | | |
