# Supplementary material for: The long noncoding RNA LUCAT1 promotes colorectal cancer cell proliferation by antagonizing Nucleolin to regulate MYC expression
Source: Cell Death Dis. 2020 Oct 23;11(10):908. doi: 10.1038/s41419-020-03095-4 (PMC7584667; doi:10.1038/s41419-020-03095-4)
Supplement: Supplementary file 14 — Supplementary Table8 [file 41419_2020_3095_MOESM14_ESM.doc]

**Supplementary Table 8. Predicted G4 forming sequence of *LUCAT1* and *MYC* promoter**

| Full length of *LUCAT1* (5'---3') and predicted G4 forming sequence of *LUCAT1* (716bp to 746bp):  AATCAACACTCCACTCAGACAATGCCCAGACCTCCAGAAACCATGTGTCAAGCTCGGATTGCCTTAGACAGGTGCAATTTAAGAACAGCTTTCATCCTCTTTTCTCTCATATTGTCACACTATGTGTTCTGACTTCTGGCTCCTTTCCTCACAAGAAGCTCACCCAGCTGGAACTCTTATGGGACCTTGGCACCAGAGACCACAAATTCCTCTTTGAAGTTTTCTAACAGCAACAATGGTATTTCTGACTTGGCTTTCTTGTATTTCTCTCACGTTAACAAAATTGGTTCAGCATCTACCATGGGCTACATGCTGAGCTACAGAGTTTCGCTCTGTCGCCCAGGCTGGAGTGCAGTGGCGCGCGATCTCGGTTCACTGCAAGCTCCACCTCCCGGGTTCACGCCATTCTCCTGCCTCAGCCTCCTGAGTAGCTGGGACTACAGGCGCCCGCCACCACACCCAGGAATCCAACTTGCTGTTTGCTATCACATGTGCTATACATGCTGTTGATGAAACTGCTAAAGGGGCTGAATGTGACTGACGTCTTTGGAAGGATGAGACTTAGCGTGCCTGTACAGTTGTGTCCAAATGCTGTCCTCATCTCCCAATGAAAAGGAACAAAACCCATCAGAAGATGTCAGAAGATAAGGATTTTTGTCCTGATGCTACACTTACCAGCTGTCCCTCAGTGTTCTACTTCTTAAAAAAAGAGAGATGGATAAACAGAGGCAACCCGAGGATAAAGGCCTTGCTCAGTGTCACACATTTCAGTCACTAAATAAGACACAATGGATGCCAGTATTCTCATCCCCTCACAAATAAAGAGCCTTCAAGCTCTTGCAGTCAACAAGAACTTTTGGAATGATTTCACTGCCTGAAAAGGCAGATAC  *MYC* Promoter (-1000 bp to 0 bp) and predicted G4 forming sequence of *MYC* promoter (-141bp to -114bp):  AGCCCGAGACTGTTGCAAACCGGCGCCACAGGGCGCAAAGGGGATTTGTCTCTTCTGAAACCTGGCTGAGAAATTGGGAACTCCGTGTGGGAGGCGTGGGGGTGGGACGGTGGGGTACAGACTGGCAGAGAGCAGGCAACCTCCCTCTCGCCCTAGCCCAGCTCTGGAACAGGCAGACACATCTCAGGGCTAAACAGACGCCTCCCGCACGGGGCCCCACGGAAGCCTGAGCAGGCGGGGCAGGAGGGGCGGTATCTGCTGCTTTGGCAGCAAATTGGGGGACTCAGTCTGGGTGGAAGGTATCCAATCCAGATAGCTGTGCATACATAATGCATAATACATGACTCCCCCCAACAAATGCAATGGGAGTTTATTCATAACGCGCTCTCCAAGTATACGTGGCAATGCGTTGCTGGGTTATTTTAATCATTCTAGGCATCGTTTTCCTCCTTATGCCTCTATCATTCCTCCCTATCTACACTAACATCCCACGCTCTGAACGCGCGCCCATTAATACCCTTCTTTCCTCCACTCTCCCTGGGACTCTTGATCAAAGCGCGGCCCTTTCCCCAGCCTTAGCGAGGCGCCCTGCAGCCTGGTACGCGCGTGGCGTGGCGGTGGGCGCGCAGTGCGTTCTCGGTGTGGAGGGCAGCTGTTCCGCCTGCGATGATTTATACTCACAGGACAAGGATGCGGTTTGTCAAACAGTACTGCTACGGAGGAGCAGCAGAGAAAGGGAGAGGGTTTGAGAGGGAGCAAAAGAAAATGGTAGGCGCGCGTAGTTAATTCATGCGGCTCTCTTACTCTGTTTACATCCTAGAGCTAGAGTGCTCGGCTGCCCGGCTGAGTCTCCTCCCCACCTTCCCCACCCTCCCCACCCTCCCCATAAGCGCCCCTCCCGGGTTCCCAAAGCAGAGGGCGTGGGGGAAAAGAAAAAAGATCCTCTCTCGCTAATCTCCGCCCACCGGCCCTTTATAATGCGAGGGTCTGGACGGCTGAG |
| --- |
