# Supplementary material for: The long noncoding RNA LUCAT1 promotes colorectal cancer cell proliferation by antagonizing Nucleolin to regulate MYC expression
Source: Cell Death Dis. 2020 Oct 23;11(10):908. doi: 10.1038/s41419-020-03095-4 (PMC7584667; doi:10.1038/s41419-020-03095-4)
Supplement: Supplementary file 15 — Supplementary Table9 [file 41419_2020_3095_MOESM15_ESM.doc]

**Supplementary Table 9.** **Plasmids for promoter reporter system and overexpression.**

| **1. pGL3-basic promoter reporter system** | | |  | |
| --- | --- | --- | --- | --- |
| Name | Insert length | Mutant sequence 5’---3’ | | |
| h-pGL3-basic-*MYC*-WT | 1000bp | NA | | |
| h-pGL3-basic-*MYC*-MUT | 1000bp | “CCTTCCCCACCCTCCCCACCCTCCCCA” to “TTAATTAATTTAATTAATTTAATTAAT” | | |
| **2. Overexpression plasmid** | | |  | |
| Name | Insert length | Mutant sequence 5’---3’ | |  |
| pcDNA3.1(+)-*LUCAT1*-WT | 890bp | NA | |  |
| pcDNA3.1(+)-*LUCAT1*-MUT | 890bp | “GGATAAACAGAGGCAACCCGAGGATAAAGG” to “AATATTTTTATAATTTTTTATAATA” | |  |
